# Editorial: Drug discovery from natural sources: Animal venoms, plants, bacteria and fungi

**DOI:** 10.3389/fmolb.2022.998676

**Published:** 2022-09-02

**Authors:** Hang Fai Kwok

**Affiliations:** ^1^ Institute of Translational Medicine, Faculty of Health Sciences, University of Macau, Taipa, China; ^2^ Cancer Centre, Faculty of Health Sciences, University of Macau, Taipa, China; ^3^ Department of Biomedical Sciences, Faculty of Health Sciences, University of Macau, Taipa, China

**Keywords:** natural bioactive compounds, phytochemicals, diseases, diagnosis, precision treatments, targeted therapies

Through several thousand million years of adaptive evolution, venomous animal species, toxic plants, and microorganisms have developed a large group of high-affinity and stable natural-based biomolecules, which have now been isolated and identified. Some of these biomolecules can specifically bind to human receptors/ligands, which means they have a great potential to be used as novel prototype drug candidates for disease treatments such as hypertension, cancer, diabetes, drug-resistant bacterial infections etc. In the last 2 decades, a lot of studies have been focused on certain types of specific molecular targets on cells, which can be recognized by natural-based biomolecules, to unveil their use in different aspects of clinical prognosis and therapeutic applications. However, many natural-based prototype drugs entering clinical trials are still struggling because their modes of action are not fully identified. Thus, we must do more lab-based and preclinical work toward this research direction.

In this Research Topic, we brought together leading researchers to exchange and share their findings on the hot topics of novel identified venom-based and plant-derived natural biomolecules with potent pharmacological activity and their corresponding molecular mechanisms to fulfil the gap which is hindering their potential use as novel prototype drug candidates for disease treatments such as cancer, hypertension, diabetes, anti-bacterial infection, and neurological disorders.

An important aspect of this Research Topic is to unleash the anticancer potentials of the natural biomolecules such as cytotoxins and peptides. Chong et al. identified two cytotoxins from *Naja sumatrana* (NS-CTX) and *Naja kaouthia* (NK-CTX), which were highly potent in inhibiting the growth of lung cancer cell lines selectively. Chong’s research team further concluded that these cobra cytotoxins have such potential; however, their highly evolved and diet-adapted cytotoxic nature might have limited the intended application as a safe and effective drug. From their study, the data underscores the need for a comprehensive, fundamental investigation that addresses the selectivity and cell death mechanism of venom proteins in the quest for novel anticancer agents. However, the anticancer potential of these toxins could be further improved through structural modification to deliver a more specific, molecularly targeted cancer therapy in the future. In another research led by de Avelar Júnior et al., their findings confirmed that the peptide named LyeTx I-b is an excellent potential candidate for combined chemotherapy to treat breast cancer. In addition, de Avelar Júnior’s team also indicated that *in vivo* studies are essential to validate the use of LyeTx I-b as a therapeutic drug candidate, alone or combined with cisplatin. With the concept of using natural biomolecule and FDA-approved drugs as combined cancer therapy, Caballero et al. study provided another example to show two component prototypes named SF3 and SF4 for development as new drugs for glioblastoma treatment. Their findings stimulate studies to use these compounds in combination therapy with a rapamycin-like activity. Caballero’s team concluded that future studies would be conducted to characterize, synthesize the molecules, and evaluate the efficacy and safety in preclinical models.

Cardiovascular disorders such as ischemic stroke and thrombotic disease have been considered major causes of death worldwide. Yang et al. carried out extensive metabolomic analysis. They found that 26 biomarkers can be regulated by folic acid via metabolic pathways of amino acid metabolism, carbohydrate metabolism, fatty acid metabolism, citrate cycle, and pyruvate metabolism, which may be the potential therapeutic targets of folic acid against ischemic stroke. Folic acid, as a natural anti-fibrosis agent, has significant activity in protecting against middle cerebral artery occlusion-induced rat ischemic stroke model by delaying pathological development, reversing the metabolic biomarkers, and mainly regulating the perturbation in amino acid metabolism, carbohydrate metabolism, fatty acid metabolism, citrate cycle, and pyruvate metabolism. The data from this study also indicated that this integrated metabolic biomarker screening platform could better understand the therapeutic effect and mechanism of drugs. In another study, Liu et al. identified a novel fibrinolytic protein named EPF3, with strong fibrinolytic activity, which was purified from *Pheretima vulgaris*. Liu’s research team constructed a three-dimensional structure of EPF3, and further performed a molecular docking analysis. They predicted that EPF3 could directly interact with antithrombotic target proteins, which was further confirmed by further studies. In conclusion, this antithrombotic mechanism of EPF3 was clarified to be outstanding direct fibrinolysis, fibrinogenolytic activity, and certain plasminogen activation. EPF3 possesses the potential to be developed into a promising antithrombotic agent.

Alpha-amylase is widely exploited as a drug target for preventing postprandial hyperglycemia in diabetes and other metabolic diseases. However, inhibition of this enzyme by plant-derived pregnanes is not fully understood. Herein, Ogunyemi and his research team (Ogunyemi et al.) employed *in vitro*, *in silico* and *in vivo* studies to provide further insights into the alpha-amylase inhibitory potential of selected pregnane-rich chromatographic fractions and four steroidal pregnanes phytochemicals (SPPs). Their study showed that steroidal pregnane and pregnane glycosides were derived from Gongronema latifolium Benth. Might be exploited as inhibitors of pancreatic alpha-amylase as an oral policy for impeding postprandial blood glucose rise for the treatment of diabetes.

Antimuscarinic drugs are well-known agents for a kind of neurological disorder that have earned their place in the management of overactive bladder (OAB). The study conducted by Zapała et al. aimed to investigate whether phytomedicine extracts from *Lindera aggregata* root, *Equisetum arvense* stem, and *Crateva nurvala* stem bark (Urox^®^) would reverse RA-induced changes in several cystometric and biochemical parameters characteristic of bladder overactivity and to thus check if this herbal supplement could be a reasonable strategy, as a future pharmacological treatment for patients with OAB. Zapała’s results concluded that Urox^®^ were potent in reversing RA-induced changes in several cystometric and biochemical parameters that are determinants of OAB. Furthermore, they observed no effects on basic cardiovascular parameters and daily urine output, which may promote the initiation of the analysis of its safety in humans.

Finally, a review article by Amorim-Carmo et al. described some strategies to optimize scorpion AMPs. Amorim-Crmo and his team addressed the primary sequence, biotechnological potential, and characteristics that should be considered when developing an AMP derived from scorpion venoms. In addition, this review contributes toward improving the understanding of rationally designing new molecules, targeting functional AMPs that may have a therapeutic application.

Overall, the research and review articles included in this Research Topic expand our knowledge of the latest studies in venom-based and plant-derived natural biomolecules. These bioactive molecules with potent pharmacological activities have their corresponding molecular mechanisms to fill the gap hindering the potential use as novel prototype drug candidates for disease treatments. In addition, the state-of-the-art analytic techniques and research platforms that have been widely adopted in the last half decade will continue to contribute to future studies in the field of drug discovery from natural sources.

